# Street ketamine use and differential risk of suicidality among adults in Taiwan

**DOI:** 10.1186/s12954-025-01308-7

**Published:** 2025-09-29

**Authors:** Yen-Chun Kuo, Sheng-Chang Wang, Chuan-Yu Chen

**Affiliations:** 1 Linsen Chinese Medicine and Kunming Branch, Taipei City Hospital, Taipei, Taiwan; 2 Center for Neuropsychiatric Research, National Health Research Institutes, 35 Keyan Road, Zhunan, Miaoli County Taiwan; 3 Department of Psychiatry, Cardinal Tien Hospital, New Taipei City, Taiwan; 4 Institute of Public Health, Medical Building II, Rm 210, National Yang Ming Chiao Tung University, No. 155, Sec. 2, Linong Street, Taipei, 11221 Taiwan; 5 Department of Epidemiology & Biostatistics, College of Human Medicine, Michigan State University, East Lansing, Michigan, , USA

**Keywords:** Ketamine, Suicide risk, Suicidality, Anxiety disorders, Mental health

## Abstract

**Introduction:**

The misuse of ketamine, a dissociative anesthetic, has surged sharply in popularity across East and Southeast Asia and Oceania. This study aimed to evaluate the association between recent patterns of ketamine use and the risk of suicide in northern Taiwan.

**Methods:**

Illicit ketamine users were recruited through network-based sampling from 2015 to 2017, along with a comparison group of individuals who never used ketamine. Data on sociodemographics, drug use history, psychiatric disorders, and suicide risk were collected by trained psychiatric nurses. Participants were categorized into three groups: non- (*n* = 132), past (no ketamine use for ≥ 1 year; *n* = 80), and recent users (use in the past year; *n* = 167). Suicide risk, assessed by six items in the Mini-International Neuropsychiatric Interview, was classified as “no,” “low,” or “middle/high.” Polytomous logistic regression and multivariate analyses were applied for risk estimation.

**Results:**

Street ketamine users disproportionately experienced higher mental health disorders (e.g.,major depressive disorder), disadvantaged socioeconomic status, involvement in legal issues, and lifetime suicide attempts (25%). The prevalence of middle to high suicide risk was notably higher among recent (10.8%) and past (5.0%) ketamine users, compared with people who had no ketamine use history. Past-month suicidality was four times higher in recent users (95% CI 1.35–12.27) and 3.4 times higher in past users (95% CI 1.01–11.45). Excess risk associated with recent ketamine use manifested exclusively in suicidal ideation.

**Discussion and conclusions:**

Illicit ketamine use is strongly linked to increased suicidality, especially among recent users. Even past users remain at higher risk, highlighting the importance of integrating suicide risk assessments and targeted interventions in community-based alcohol and drug services.

**Supplementary Information:**

The online version contains supplementary material available at 10.1186/s12954-025-01308-7.

## Background

Ketamine has experienced a sharp surge in popularity in East and Southeast Asia and Oceania over the past decade, particularly among young people and attendees of raves or parties [1, 2]. In Taiwan, ketamine has emerged as one of the top three illegal drugs since the early twentyfirst century. Household surveys indicate a steady rise in prevalence, increasing from 0.24% in 2005 to 0.39% in 2014 and 0.40% in 2018 [3–5]. Non-medical use of ketamine is linked to a wide range of significant psychological and physical health issues, including impulsivity, cognitive impairment, dissociative symptoms, delusions, and lower urinary tract problems [6–8]. A study involving 39,178 recreational ketamine users from 2009 to 2016 reported a 3-year standardized mortality ratio (SMR) of 4.9 for all causes of death, underscoring the severe health risks associated with its use [9]; the risks are even more pronounced in suicide, with the SMR reaching 13.4 overall and rising to 31 for female users [9]. Supporting this, data from Hong Kong demonstrate that ketamine use carries the highest substance use disorder-related risk for self-harm or suicide, with a hazard ratio of 16.4 [10].

Substance use has long been recognized as a major risk factor for suicidality, increasing vulnerability to suicidal thoughts, attempts, and deaths [11, 12]. A comprehensive meta-analysis further reinforces this connection, demonstrating a strong association between substance use disorders and various forms of suicidality [13]. Among individuals who use drugs without treatment, suicidality rates are alarmingly high—recent data indicate that 23.6% experience suicidal thoughts, while 6.7% have attempted suicide within the past year [14]. However, the relationship between substance use and suicidality varies depending on the type of substance [15]. Distinct underlying mechanisms of the specific substance for different suicidal behaviors were noted. For example, marijuana use is more strongly linked to suicide attempts than ideation [16]. These findings highlight the critical need to examine the substance-specific relationship with suicidality and develop targeted intervention strategies to address the complex interplay between substance use and suicidality in diverse populations [15].

The three-step theory (3ST) of suicide provides a valuable framework for understanding the relationship between substance use and the progression from suicidal ideation to attempts. This theory identifies four key factors—pain, hopelessness, connectedness, and capability for suicide—as critical in this transition [17]. Disadvantaged conditions or stressful life events, such as poverty or incarceration, can create psychological pain, which, when coupled with hopelessness, fosters a strong suicidal desire. When this pain outweighs an individual’s sense of connectedness, suicidal desire intensifies, and the presence of capability—such as access to lethal means or reduced fear of death—can lead to a suicide attempt [17–19]. Understanding the intricate interplay of factors driving suicidality—including thoughts, planning, attempts, and deaths—across different substances is therefore crucial for designing targeted prevention strategies.

The relationship between ketamine use and the key factors of 3ST is uniquely complex. The heightened suicide risk among recent ketamine users likely stems from a combination of neurocognitive deficits, psychological distress, and social adversities [6, 20]. Chronic ketamine use is associated with significant impairments in cognitive control, including impulsivity and poor response inhibition [8, 21]. These deficits lower the threshold for transitioning from suicidal ideation to an actual suicide attempt, as observed in individuals with other substance use disorders and adolescents using cannabis or illicit drugs [22, 23]. Impaired cognitive control heightens vulnerability to impulsive behaviors, making recent ketamine users more susceptible to self-harm and suicide during moments of crisis. In addition to these neurocognitive challenges, recent ketamine users often face ongoing legal and social stressors [1, 21]—such as involvement with the criminal justice system, unemployment, and social isolation—that can exacerbate feelings of hopelessness and increase the likelihood of suicidal behavior [20, 24, 25].

Exposure to life stressors may drive certain individuals to self-medicate with ketamine to achieve transient relief from psychological distress [26, 27]. However, sustained use may reinforce maladaptive coping mechanisms and increase the risk of dependency and suicide [10, 26]. The use of street drugs frequently entails exposure to potentially lethal means (e.g., overdose) [28, 29], which may increase individuals’ knowledge of and familiarity with suicide methods, thereby enhancing their acquired capability for suicide. A previous study further highlights the role of suicide capability in linking substance use frequency to suicide attempts, as risky behaviors associated with substance use may increase exposure to trauma or bodily harm, reinforcing the desensitization to injury and death [30]. Furthermore, the psychedelic effects of ketamine may impair individuals’ perception and cognitive functioning [6], while its analgesic properties could diminish the fear of pain and death [31] — factors that may facilitate the transition from suicidal ideation to actual attempts. Paradoxically, ketamine’s pharmacological action as an N-methyl-D-aspartate (NMDA) receptor antagonist shows initial promise in reducing suicidal ideation in patients with major depressive disorder and anxiety disorders [32–35]. These dual effects implicate the importance of contextualizing ketamine use within its social and clinical settings, highlighting the need to carefully weigh its clinical benefits against its potential for misuse and associated risks, particularly in populations vulnerable to its adverse effects.

While ketamine remains a significant substance of concern due to its non-medical use in many Asian regions [1, 4, 10], its growing therapeutic applications in mental health—particularly for treatment-resistant depression—in Western healthcare settings present a complex duality [33, 34]. Existing research on the elevated suicide risk associated with non-medical ketamine use has largely focused on individuals involved with criminal justice systems or healthcare services [9, 10]. Similarly, studies emphasizing ketamine’s beneficial effects on suicidal ideation have primarily examined treatment-seeking individuals with severe depressive disorders [32, 33]. These ascertainment sources likely capture distinct population subsets, reflecting differences not only in the prevalence and history of mental disorders but also in exposure to disadvantaged conditions and stressful life events. However, the focus on institutionalized populations—such as those who are incarcerated or hospitalized—limits the generalizability of findings to ketamine users with no prior treatment history in community settings, many of whom use the drug recreationally. Furthermore, the reliance on death records as a primary data source creates significant gaps in understanding how the risk of suicidal indicators evolves with ketamine involvement, particularly among hidden or underrepresented populations in the community. Addressing these limitations is crucial for gaining a more comprehensive understanding of the relationship between ketamine use and suicidality.

To address the existing knowledge gap and deepen our understanding of the relationship between ketamine use and suicidality in real-world settings, we conducted a community-based study in Taiwan, employing a social network approach to recruit ketamine users. The objectives of this study were to evaluate whether the level of suicide risk and past-month suicidality is associated with a history of ketamine use and to explore whether these associations differ across specific suicidality indicators.

## Methods

### Study population and data source

Two groups of participants were included in this study: ketamine users and ketamine non-users.

For ketamine users, participants were eligible if they were aged 20 years or older and had a history of street ketamine use. They were enrolled using snowball sampling in the Taipei and Taoyuan metropolitan areas. The process began with recruiting the initial participants, the so-called “seeds”, through advertisements posted in the collaborating hospitals (Far Eastern Memorial Hospital and En Chu Kong Hospital), or via referrals from individuals seeking medical help for ketamine use problems. Upon completing the study procedure, the participants were asked to refer the next wave of respondents from their acquaintances who met the same eligibility criteria. For each successful referral, an additional NTD 300 (approximately USD 10) was provided to the introducers. Through this chain-referral process, the study recruited 50 initial seeds, 19 of whom provided further referrals. A total of 250 ketamine users, including the seeds, were enrolled.

As to ketamine non-users, individuals were eligible if they were aged 20 years or older and had no history of illicit drug use. Recruitment for this group was conducted exclusively through advertisements, and a total of 132 ketamine non-users were enrolled.

### Study procedure

All participants provided written informed consent prior to undergoing the study procedures, which were performed in the collaborating hospitals. Given that ketamine is a Schedule III controlled drug and its use may involve legal liability, all participants were allowed to sign the informed consent under pseudonyms. Four trained psychiatric nurses with over 15 years of research experience conducted clinical assessments covering demographics, drug use and treatment history, psychiatric diagnoses, and suicidality. Upon completion of the assessments, they would receive a gift voucher valued at NTD 2,000 (approximately USD 70). The study protocol was approved by the Institutional Review Board of the National Health Research Institutes, Miaoli, Taiwan (IRB EC1060101). The study was conducted between August 2015 and July 2017.

### Measures

Dependent variables: this study evaluated two key outcome variables—overall suicide risk and past-month suicidality. These were measured using the suicidality module of the Mini International Neuropsychiatric Interview (MINI), a validated and widely utilized diagnostic tool [36]. The MINI, translated into Mandarin by the Taiwan Society of Psychiatry [37], has been extensively employed in both clinical and research settings due to its reliability and ease of administration. This allowed for a standardized assessment of suicidal thoughts, plans, and behaviors in a non-clinical setting.

**Fig. 1:**
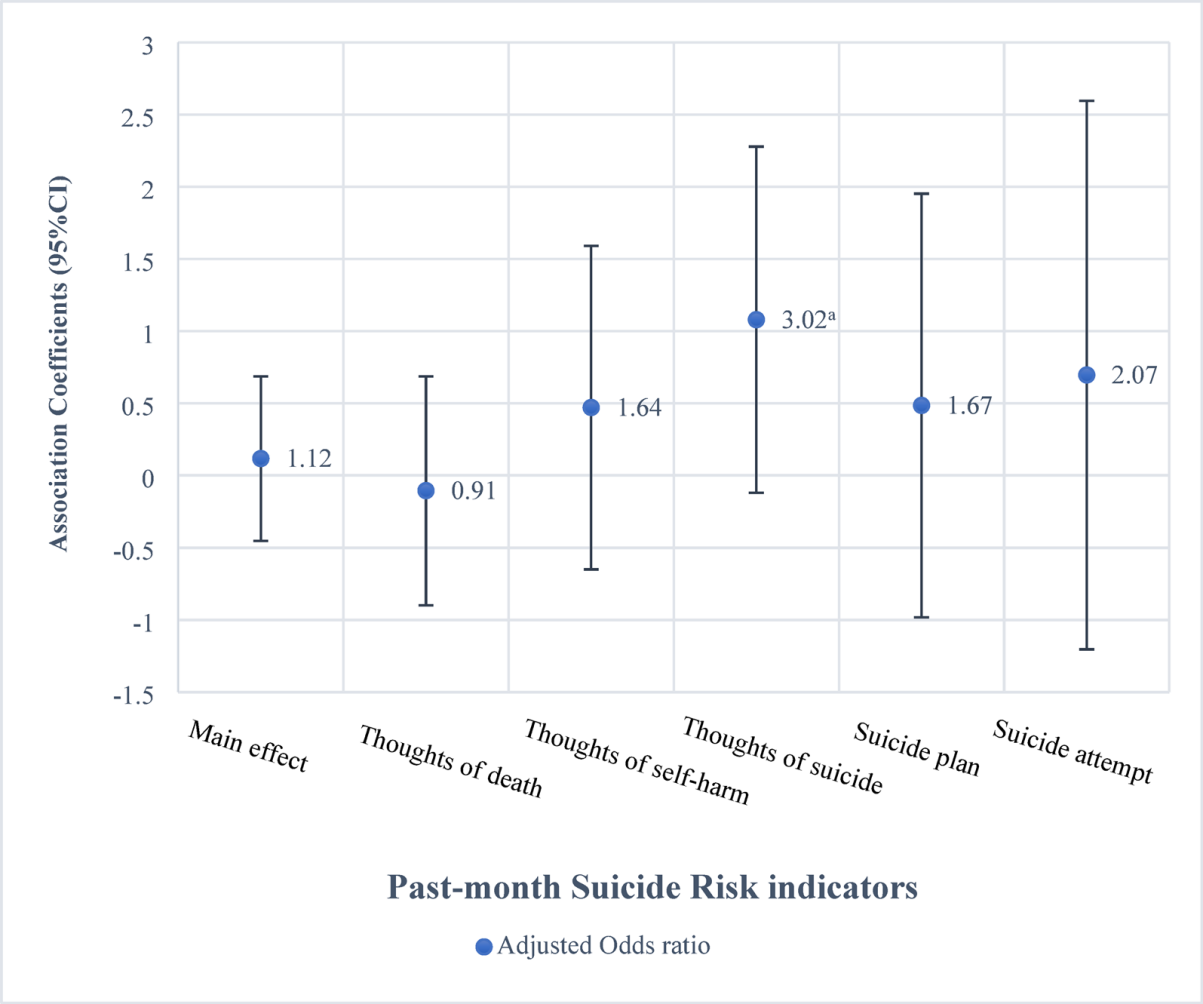
Estimated association linking ketamine use with past-month suicide risk by the GLM/GEE analysis. Note. Estimated regression coefficients and 95% CI were obtained from GLM/GEE. Adjusted odds ratio from the model including covariates: gender, marital status, age, educational attainment, employment, legal status, lifetime suicide attempt, and history of mental problems. ^a^*p*=0.071

The suicidality module used in this study consisted of six indicators, five of which assessed suicidal behaviors and thoughts occurring in the past month. These included: (S1) “*Have you thought that you would be better off dead or wished you were dead in the past month?*“; (S2) “*Have you wanted to harm yourself in the past month?“;* (S3) “*Have you thought about suicide in the past month?*“; (S4) “*Have you had a suicide plan in the past month?*“; and (S5) *“Have you attempted suicide in the past month?*” Additionally, a lifetime indicator (S6) assessed whether participants had ever made a suicide attempt. Based on Sheehan et al.‘s (1998) criteria, overall suicide risk was categorized as low, moderate, or high. Since lifetime suicide attempts (S6) are less likely to be affected by current ketamine use, analyses requiring temporal ordering—such as those in Table 3; Fig. 1—were restricted to past-month suicidality measures (S1–S5). This approach allowed for a clearer assessment of the temporal relationship between past-year ketamine exposure and past-month suicidality.

Independent variables: among participants with a history of ketamine use, 247 provided complete clinical data and were further categorized into two groups: (i) past users (*n* = 80), referring to individuals who had not used ketamine in the past year, and (ii) recent users (*n* = 167), referring to individuals who had used ketamine within the past year. This categorization allowed for a clear distinction between individuals who had used ketamine in the past year and those who had not.

Covariates: sociodemographic information collected in the study included gender, age, marital status, educational level, employment status, and legal status. Educational attainment was categorized as “middle school or below” versus “high school or above.” Employment status, initially recorded as “none,” “part-time job,” or “full-time job,” was reclassified into a binary variable: “unemployed (yes)” or “unemployed (no).” Legal involvement was assessed by inquiring whether participants were currently under legal protection, custody, parole, deferred prosecution, on trial, or serving a sentence. Mental disorders were evaluated using the Structured Clinical Interview for DSM-IV-TR Axis-I Disorders (SCID-I), focusing on sections A to F [38]. Trained research nurses conducted the assessments to ensure accuracy and consistency. A positive history of major depressive disorder (MDD), anxiety disorders, or alcohol use disorder was defined as cases in which the onset of the illness occurred at least one year before the interview.

### Data analysis

Descriptive analyses were conducted by stratifying participants into three groups based on their ketamine use experience, with comparisons performed using Chi-square tests and Fisher’s exact tests. To assess the relationship between recent or past ketamine use and suicide risk levels (categorized as no risk, low risk, or middle/high risk), polytomous logistic regression was used, with the independent variable—ketamine use experience—categorized as none, past, or recent (*n* = 379). Factors associated with low and middle/high suicide risk were analyzed, and the results were presented as relative risk ratios (RRRs) with corresponding 95% confidence intervals (CIs).

Recognizing that the five past-month suicide risk indicators are interrelated, we turned to multivariate analyses to examine the relationship between ketamine exposure and suicidality. A multivariate response profile regression approach, based on Generalized Linear Models (GLM) with Generalized Estimating Equations (GEE), was used to account for potential intra-individual dependency among these indicators. This statistical method, initially described by Liang, Zeger, and Qaqish [39] and applied in subsequent studies [40, 41], allowed for robust estimation of associations while considering the correlated nature of suicidality indicators within individuals.

Recent ketamine exposure was defined as use within the past year, while suicidality propensity was assessed based on past-month experiences of five suicidal indicators to ensure a clear temporal sequence. To evaluate the relationship between ketamine use (recent vs. past) and suicidal propensity, the common slope model was initially applied, assuming consistent effects of ketamine exposure across all five suicide risk indicators. This analysis was conducted both with and without covariate adjustments. Finally, for participants who had used ketamine at least once in their lifetime (*n* = 246), the single slope model was utilized to examine specific associations between ketamine use and each individual suicide risk indicator. Interaction effects were captured by introducing product terms. Variance estimators were calculated using the Generalized Estimating Equations working correlation structure with robust variance estimation to account for potential intra-individual dependencies. All statistical analyses were conducted using STATA SE version 15.0.

## Results

Compared to non-users (see Table 1), individuals with ketamine experience were more likely to be male, unmarried, have lower educational attainment, be unemployed, face legal issues, and report a history of MDD or alcohol use disorder. The prevalence of middle/high suicide risk was significantly higher among recent ketamine users (10.8%) and past users (5.0%) compared to the ketamine-non group (1.5%). Additionally, recent ketamine users demonstrated the highest prevalence of past-month self-harm thoughts and suicidal thoughts among all subgroups.

**Table 1:** Selective characteristics of adults in metropolitan areas by ketamine use experience (n = 379)

Variables	Ketamine use experience						*P* value
	Ketamine-none (n = 132)		Ketamine-past (n = 80)		Ketamine-recent (n = 167)		
	N	%	N	%	N	%	
Sociodemographic background							
Gender							0.004
Males	92	69.7	61	76.3	143	85.6	
Females	40	30.3	19	23.8	24	14.4	
Marital status							0.010
Single, divorced …	81	61.4	62	77.5	126	75.5	
Married or cohabitated	51	38.6	18	22.5	41	24.6	
Age (years old)							0.091
20–29	70	53.0	46	57.5	112	67.1	
30 or above	62	47.0	34	42.5	54	32.3	
Educational attainment							0.000
Middle school or below	13	9.9	40	50.0	97	58.1	
High school or above	119	90.2	40	50.0	70	41.9	
Unemployment							0.004
No	122	92.4	64	80.0	132	79.0	
Yes	10	7.6	16	20.0	35	21.0	
Involvement in legal issues (current)							0.000^a^
No	132	100.0	65	81.3	111	66.5	
Yes	0	0	15	18.8	56	33.5	
Mental problems							
Prior history of major depressive disorder							0.023
No	109	82.6	59	73.8	122	73.1	
Yes	23	17.4	19	23.8	45	27.0	
Missing	0	0	2	2.5	0	0	
Prior history of anxiety disorders							0.596
No	115	87.1	64	80.0	137	82.0	
Yes	16	12.1	14	17.5	26	15.6	
Missing	1	0.8	2	2.5	4	2.4	
Prior history of alcohol use disorder							0.000
No	99	75.0	23	28.8	51	30.5	
Yes	31	23.5	55	68.8	115	68.9	
Missing	2	1.5	2	2.5	1	0.6	
Suicide attempt (lifetime)	15	11.4	20	25.0	43	25.8	0.005
Suicide risk in the past month							
Thoughts of death	6	4.6	21	26.3	45	27.0	0.000
Thoughts of self-harm	2	1.5	5	6.3	18	10.8	0.006
Thoughts of suicide	1	0.8	3	3.8	18	10.8	0.001
Suicide plan	0	0	2	2.5	8	4.8	0.021^a^
Suicide attempt	0	0	1	1.3	5	3.0	0.102^a^
Suicide risk							0.000
No	114	86.4	50	62.5	105	62.9	
Low	16	12.1	26	32.5	44	26.4	
Middle/high	2	1.5	4	5.0	18	10.8	

^a^Fisher's exact test

As shown in Table 2, participants with ketamine-past experience were significantly more likely to have a low level of suicide risk compared to the ketamine-non users (RRR = 3.71; 95% CI 1.83–7.51). Recent ketamine users exhibited a markedly elevated risk of middle/high suicide risk, with a 9.77-fold increase (RRR = 9.77; 95% CI 2.21–43.13). After adjusting for covariates, the association between ketamine-past experience and low suicide risk remained robust (aRRR = 2.93; 95% CI 1.24–6.93), while the elevated risk for middle/high suicide risk among recent users became attenuated. A history of anxiety disorders (aRRR = 6.30) and involvement in legal issues (aRRR = 4.61) were strongly associated with middle/high suicide risk.

**Table 2:** Association linking ketamine use with levels of suicide risk (n = 379)

Variable	Unadjusted model (n = 379)^a^				Adjusted model (n = 379)^a,b^			
	Low (n = 86)		Middle/high (n = 24)		Low (n = 86)		Middle/high (n = 24)	
	RRR	95% CI	RRR	95% CI	aRRR	95% CI	aRRR	95% CI
Ketamine use experience (Ref: Ketamine-none)								
Ketamine-past	3.71	1.83–7.51***	4.56	0.81–25.71	2.93	1.24–6.93*	1.99	0.27–14.43
Ketamine-recent	2.99	1.59–5.61**	9.77	2.21–43.13**	2.31	1.18–5.63*	3.67	0.60–22.36
Sociodemographic characteristics								
Females (Ref: Males)	1.95	1.12–3.39*	2.24	0.91–5.54	2.29	1.16–4.51*	4.66	1.43–15.19**
Marital status (Ref: Married or cohabitated)								
Single, divorced	1.03	0.61–1.76	1.61	0.58–4.46	0.95	0.51–1.78	2.48	0.70–8.75
Age (years) (Ref: 30 or above)								
20–29	1.25	0.15–1.10	0.81	0.35–1.86	1.03	0.58–1.83	0.57	0.21–1.52
Educational attainment (Ref: High school or above)								
Middle school or below	1.87	1.14–3.06	4.75	1.90–11.87	1.44	0.79–2.62	3.46	1.12–10.69 ^*^
Unemployment (Ref: No)	1.43	0.75–2.73	3.14	1.25–7.84	1.10	0.53–2.27	2.37	0.79–7.05
Involvement in legal issues (Ref: No)	2.68	1.48–4.84**	8.17	3.39–19.69***	1.60	0.79–3.26	4.61	1.49–14.20**
Mental problems								
Prior history of major depressive disorder (Ref: No)								
Yes	3.23	1.88–5.56***	3.64	1.52–8.71**	2.38	1.29–4.37**	2.34	0.83–6.62
Prior history of anxiety disorders (Ref: No)								
Yes	5.03	2.63–9.60***	8.97	3.52–22.91***	3.82	1.86–7.84***	6.30	2.11–18.81**
Prior history of alcohol use disorder (Ref: No)								
Yes	2.01	1.21–3.35**	2.58	1.04–6.42*	1.17	0.62–2.20	1.03	0.33–3.23

*RRR* relative risk ratio, *aRRR* adjusted relative risk ratio, *CI* confidence interval, *Ref* reference group

^a^No suicide risk group as a reference

^b^Adjusted model: adjusted for sociodemographic and mental health variables

*p < 0.05, **p < 0.01, ***p < 0.001

Both the ketamine-past and ketamine-recent groups demonstrated a significantly higher risk of past-month suicidality compared to the ketamine-non group, with odds ratios of 6.16 (95% CI 2.30–16.49) and 7.05 (95% CI 2.82–17.65), respectively (see Table 3). Even after further adjustments for mental health conditions and lifetime suicide attempt history, individuals with recent ketamine use appeared to have the highest risk of overall suicidality (see Model 2C^c^ : aOR = 4.06). Finally, among individuals with ketamine experience (*n* = 246), there was no significant difference in overall suicidality risk associated with recent ketamine use. However, an elevated risk specifically emerged for “thoughts of suicide.” (aOR = 3.02; 95% CI 0.91–10.03)(see Fig. 1 and Supplementary Table S1).

**Table 3:** Multivariate analyses on ketamine consumption-associated past-month suicidal propensity (n = 379)

Variable	Past month suicidality							
	Crude (n = 379)		Model 2A^a^ (n = 378)		Model 2 B^b^ (n = 378)		Model 2C^c^ (n = 378)	
	OR	(95% CI)	aOR	95% CI	aOR	95% CI	aOR	95% CI
Ketamine use (Ref: Ketamine-none)								
Ketamine-past	6.16	2.30–16.49***	3.90	1.39–10.98*	3.88	1.34–11.21*	3.41	1.01–11.45*
Ketamine-recent	7.05	2.82–17.65***	4.29	1.53–12.02**	4.00	1.42 -11.32**	4.06	1.35–12.27*
Sociodemographic characteristics								
Females (Ref: Males)	1.26	0.65–2.43	2.06	1.01–4.18*	1.33	0.64–2.74	0.60	0.25–1.45
Marital status (Ref: Married or cohabitated)								
Single, divorced	1.63	0.85–3.14	1.75	0.89–3.44	1.47	0.72–2.98	1.40	0.67–2.91
Age (years) (Ref:30 or above)								
20–29	0.99^d^	0.57–1.74	0.73	0.41–1.31	0.76	0.41–1.40	0.87	0.45–1.66
Educational attainment (Ref: High school or above)								
Middle school or below	2.76	1.60–4.77***	1.67	0.92–3.03	1.88	0.99–3.54 ^e^	2.09	1.10–3.99*
Unemployment (Ref: No)	1.84	0.94–3.60	1.43	0.74–2.75	1.16	0.57–2.37	1.25	0.58–2.70
Involvement in legal issues (Ref: No)	4.33	2.43–7.72***	3.05	1.64–5.68***	2.39	1.17–4.88*	1.66	0.82–3.38
Mental problems								
Prior history of major depressive disorder	2.21	1.22–4.03**			1.33	0.70–2.53	0.87	0.44–1.75
Prior history of anxiety disorders	5.40	2.84–10.26***			4.80	2.39–9.65***	4.85	2.23–10.53***
Prior history of alcohol use disorder	1.12	0.95–1.32			1.02	0.51–2.01	1.12	0.55–2.30
Suicide attempt (lifetime)	9.87	5.58–17.43***					8.91	4.48–17.69***

*aOR* adjusted odds ratio, *CI* confidence interval, *Ref* reference

^a^Model 2A: adjusted for sociodemographic variables

^b^Model 2B: adjusted for sociodemographic and mental health variables

^c^Model 2C: adjusted for sociodemographic, mental health variables, and lifetime suicide attempt

^d^One observation was dropped

^e^*p* = 0.052

*p < 0.05, **p < 0.01, ***p < 0.001

## Discussion

This cross-sectional study of adults in northern Taiwan underscores the strong association between ketamine use and suicidality. Ketamine-experienced individuals demonstrated a two- to three-fold higher likelihood of low-level suicide risk, with recent users exhibiting a striking four-fold increase in middle/high-level suicide risk. Ketamine exposure was consistently linked to a four-fold higher risk of past-month suicidality, irrespective of abstinence status. Among the ketamine-experienced group, the risk was particularly elevated for “thoughts of suicide,” highlighting a critical indicator of vulnerability. Furthermore, while mental disorders were identified as significant risk factors across all suicide risk levels, low educational attainment and legal issues were uniquely associated with middle/high-level suicide risk.

Nearly one-quarter of ketamine users had experienced at least one suicide attempt in their lifetimes, a rate higher than those reported among treatment-seeking individuals with heroin use disorder (17.8%), methamphetamine dependence (15.8%), or any illegal drugs (14.2%) in Taiwan [42–44]. These findings align with a Hong Kong cohort study indicating that ketamine use carries the highest substance use disorder-related risk for self-harm or suicide (hazard ratio = 16.4), surpassing opioids (16.0) and amphetamine-related stimulants (10.30) [10]. However, the observed prevalence of lifetime suicide attempts among ketamine users in this study was lower than rates reported among regular illegal drug users in Australia (32%) and Turkey (43%) [45, 46], suggesting complex variations in vulnerability across drug-using populations. These differences may reflect variations in the likelihood of transitioning from suicidal ideation to attempts and in the risk of suicidality, shaped by cultural norms around help-seeking behaviors, stigma, and access to healthcare [47]. Furthermore, the distribution of suicide risk factors, including mental health conditions and disadvantaged socioeconomic status, likely varies significantly across subpopulations and regions. Finally, the attributable risk of psychiatric disorders in driving suicidality can differ due to variations in treatment accessibility, legal frameworks, and community support systems [48–50].

Surprisingly, ketamine-past individuals in this study demonstrated persistent suicidality despite abstaining from ketamine for over a year. This aligns with findings that reported increased depressive symptoms in both frequent and past ketamine users [51], indicating the lingering psychological and emotional impact of ketamine exposure. In addition to these neuropsychological factors, ketamine-past users in our study demonstrated significant social vulnerabilities, as evidenced by higher rates of being single or divorced, lower educational attainment, and unemployment [20, 25]. These social disadvantages may hinder the recovery process, preventing these individuals from reestablishing a sense of belonging or purpose, which are critical protective factors against suicidality. Finally, a history of suicide attempts among ketamine-past users underscores their increased capability for suicide, as described by the three-step theory of suicide. This combination of biological, psychological, and social vulnerabilities likely explains their elevated rate of suicidality. The persistence of these challenges, even after prolonged abstinence, suggests the urgent need for comprehensive interventions addressing not only substance use but also the broader social and psychological factors.

Individuals with recent ketamine use in our study exhibited significantly elevated suicide risk, particularly in “thoughts of suicide” among the five past-month suicidality indicators, compared to past users. This finding contrasts with clinical studies reporting temporary anti-suicidal effects of ketamine, likely reflecting key differences in study populations. Clinical trials frequently exclude individuals with substance use or substance use disorders [33, 34], limiting the generalizability of their findings to chronic ketamine users. The acute effects of ketamine on reducing suicidal ideation appear to be highly variable. For example, one study found that 29% of participants did not experience a reduction in suicidal ideation following ketamine treatment [34]. More concerningly, a randomized trial conducted in healthy controls reported a significant increase in depressive symptoms following ketamine administration [52]. These findings suggest that the anti-suicidal effects of ketamine are not universal and may vary substantially among individuals with chronic use patterns.

## Limitations and strengths

This study has several limitations. First, the cross-sectional design limits our ability to infer causality, highlighting the need for longitudinal studies to establish temporal and directional relationships between ketamine use and suicide risk. Second, information bias is a concern, as participants’ recall accuracy may be differentially influenced by their history of ketamine consumption or comorbid mental health disorders, potentially leading to underreporting or overreporting of suicidal behaviors. Third, although snowball sampling helped reach hidden ketamine users for this study, the sample size was relatively small. Moreover, the sample might be biased by the quality and diversity of the chain-referral process, with overrepresentation of individuals from particular social networks. Therefore, the findings should be interpreted with caution, as they might not be generalizable to the broader ketamine-using population.

Fourth, while polysubstance use is common in this population, the study did not assess the past-year use patterns of substances other than alcohol, such as opioids, methamphetamine, sedative-hypnotics, and marijuana. Given their potential impact on suicidality, future study is needed to account for these confounders. Finally, caution should be taken regarding participants’ recall bias in reporting their ketamine use patterns, which may lead to misclassification. Furthermore, this study did not collect detailed information on ketamine consumption amounts or routes of administration; therefore, the findings cannot clarify the association between specific levels of ketamine exposure and suicide risk.

Despite these limitations, this study provides valuable insights into suicidality among street ketamine users, particularly focusing on a population with minimal or no prior exposure to treatment. By examining community-dwelling adults, this research offers a unique perspective on the attributes and suicide risks associated with ketamine use, addressing gaps often overlooked in studies confined to clinical settings. Findings underscore the urgent need for proactive suicide prevention strategies tailored to this vulnerable group, particularly through early risk identification and integrated mental health interventions. This community-based approach allows for a more comprehensive understanding of the real-world impact of street ketamine use on mental health, capturing nuances that might be missed in clinical populations. Moreover, the rigor of the study is enhanced by thorough clinical assessments conducted by experienced psychiatric nurses. This methodological strength stands in contrast to many community-based studies that depend solely on self-reported data, enhancing both the validity and reliability of the findings.

Our study identified significantly heightened suicidality among ketamine-experienced individuals within a community-based population in Taiwan, indicating the urgent need for regular suicide risk screening and a thorough evaluation of the long-term consequences of ketamine use. Notably, even after cessation, former ketamine users demonstrated persistently elevated suicide risk, highlighting the importance of sustained efforts to strengthen social connectedness and provide integrated mental health care for both recent and past users. Given the enduring nature of suicide risk, interventions should not only focus on acute crises but also address long-term psychosocial rehabilitation. Expanding research across diverse settings and populations is imperative to unravel the complex interplay between ketamine use and suicidality. Such efforts will refine intervention and treatment frameworks, leading to the development of targeted, evidence-based strategies that can reduce suicide risk and improve outcomes for this vulnerable group. Policymakers and healthcare providers must prioritize integrating mental health support and suicide prevention programs into alcohol and drug services while promoting initiatives that address the broader social determinants of health [20, 26].

## Supplementary Information

Below is the link to the electronic supplementary material.

Supplementary Material 1

## Data Availability

No datasets were generated or analysed during the current study.
