# Supplementary material for: Street ketamine use and differential risk of suicidality among adults in Taiwan
Source: Harm Reduct J. 2025 Sep 29;22:153. doi: 10.1186/s12954-025-01308-7 (PMC12482612; doi:10.1186/s12954-025-01308-7)
Supplement: Supplementary file 1 — Supplementary Material 1 [file 12954_2025_1308_MOESM1_ESM.docx]

# **Supplementary**

| Table S1. Estimated association linking recent ketamine use with past-month suicide risk by the GLM/GEE analysis (n=246) | | | | | | | | | | | | | | | | |  |
| --- | --- | --- | --- | --- | --- | --- | --- | --- | --- | --- | --- | --- | --- | --- | --- | --- | --- |
| Suicidal indicators |  | | | Recent ketamine use^a^ | | | | | | | | | | | | | |
|  | Past-month suicide propensity  Main effect | | | | | | Interaction with each indicator | | |  | Main + Interaction | | | | | | |
|  | | aOR^b^ | P-value | | | (95% CI) | aOR^c^ | P-value |  | | aOR^d^ | | P-value | | (95% CI) | | |
| Overall suicidality |  | 1.21 | 0.621 | | | (0.57-2.58) | 0.91 | 0.821 |  | |  | | |  | |  | |
| Thoughts of death |  |  |  | |  | | Ref | - |  | | 0.91 | 0.821 | | | (0.41-2.01) | | |
| Thoughts of self-harm |  |  |  | |  | | 1.80 | 0.244 |  | | 1.64 | 0.385 | | | (0.54-5.03) | | |
| Thoughts of suicide |  |  |  | |  | | 3.31 | 0.031 |  | | 3.02 | 0.071 | | | (0.91-10.03) | | |
| Suicide plan |  |  |  | |  | | 1.83 | 0.390 |  | | 1.67 | 0.492 | | | (0.39-7.25) | | |
| Suicide attempt |  |  |  | |  | | 2.26 | 0.375 |  | | 2.06 | 0.454 | | | (0.31-13.80) | | |

Note.

^a^ Relative to past ketamine use (n= 80).

^b^ aOR=adjusted odds ratio from the common slope model adjusted for gender, marital status, age, educational attainment, employment, legal status, lifetime suicide attempt, and past history of mental problems.

^c^ aOR=adjusted odds ratio for the model with interaction term, with simultaneous adjustment for gender, marital status, age, educational attainment, employment, legal status, lifetime suicide attempt, and past history of mental problems.

^d^ aOR=adjusted odds ratio between recent ketamine use and each suicide indicator, with simultaneous adjustment for gender, marital status, age, educational attainment, employment, legal status, lifetime suicide attempt, and past history of mental problems.
